# Corrigendum to: TIPE1 suppresses the invasion and migration of breast cancer cells and inhibits epithelial-to-mesenchymal transition primarily via the ERK signaling pathway

**DOI:** 10.3724/abbs.2024177

**Published:** 2024-12-25

**Authors:** Shusheng Qiu, Wei Hu, Qiuhong Ma, Yi Zhao, Liang Li, Yu Ding

Acta Biochimica et Biophysica Sinica 2019, 51(10): 1008–1015.

https://doi.org/10.1093/abbs/gmz099

In the original version of this manuscript, an error was found in Figure 2A and Figure 3B respectively. The correct figures are shown as follows. The authors apologize for the error.

**Figure:**
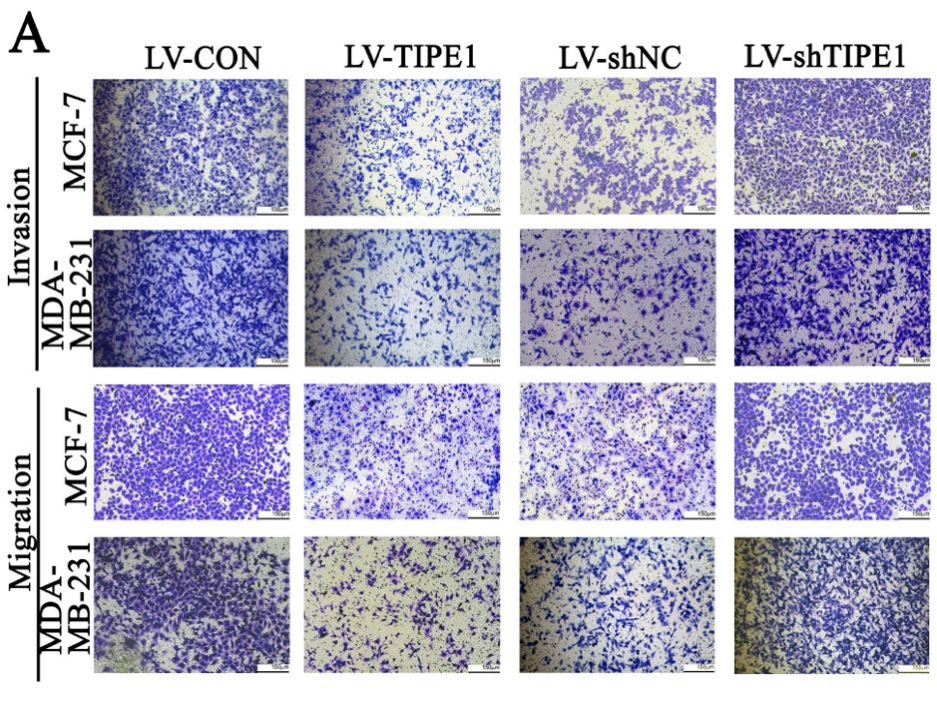
Figure 2 TIPE1 inhibited the invasion and migration of breast cancer cells

**Figure:**
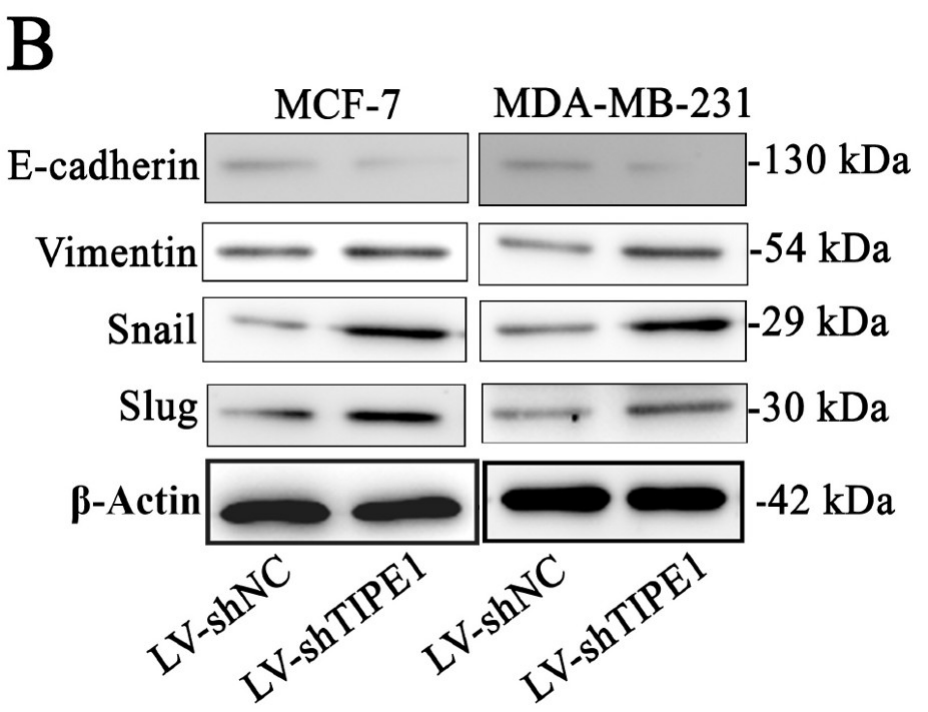
Figure 3 TIPE1 suppressed the EMT phenotype in breast cancer cells

